# Local anesthetic ropivacaine inhibits non-small cell lung cancer progression by modulating the hsa_circ_0001320/miR-518a-5p axis

**DOI:** 10.1186/s41065-025-00514-4

**Published:** 2025-07-25

**Authors:** LiQun Cheng, RongRong Shen, Zhen Ma, Yuan Guo, Yi Peng

**Affiliations:** 1 Department of Anesthesiology, Shanghai Children’s Medical Center, Shanghai Jiaotong University School of Medicine, Shanghai City, 200127 China; 2 Department of Anesthesiology, Longhua Hospital Shanghai University of Traditional Chinese Medicine, No.725, Wanping South Road, Xuhui District, Shanghai City, 200032 China

**Keywords:** Ropivacaine, Hsa_circ_0001320, miR-518a-5p, Non-small cell lung cancer

## Abstract

**Objective:**

This study investigated the possible mechanisms by which ropivacaine influences the progression of non-small cell lung cancer (NSCLC).

**Methods:**

Plasmid vectors or oligonucleotides interfering with circ_0001320 or miR-518a-5p were transfected into ropivacaine-treated NSCLC cells, and circ_0001320 and miR-518a-5p levels were detected by RT-qPCR. Cell proliferation was assessed using CCK-8, apoptosis via flow cytometry, and cell migration and invasion through Transwell assays. Finally, the binding sites of circ_0001320 and miR-518a-5p were verified by the bioinformatics website CircInteractome and dual luciferase reporter gene assay.

**Results:**

Exposure to ropivacaine resulted in the suppression of NSCLC cell proliferation, migration, invasion, and angiogenesis, while inducing apoptosis. Ropivacaine elevated circ_0001320 expression. Circ_0001320 downregulation resulted in a reduced efficacy of ropivacaine in inhibiting NSCLC progression. Interestingly, circ_0001320 targeted miR-518a-5p and inhibited its expression. It was possible to limit the effects of downregulation of circ_0001320 on NSCLC progression by downregulating miR-518a-5p.

**Conclusion:**

Ropivacaine inhibits NSCLC progression via the circ_0001320/miR-518a-5p axis.

## Introduction

Among the respiratory system cancers, lung cancer is the most common with the highest mortality and morbidity rates [1, 2]. The majority of lung cancer cases are non-small-cell lung cancers (NSCLC), such as squamous cell carcinomas, neuroendocrine tumors, large cell carcinomas, and lung adenocarcinomas [3–5]. Despite recent advances, the clinical efficacy of current treatments for NSCLC remains unsatisfactory due to its high risk of relapse and metastasis [6, 7]. The discovery of more effective anti-NSCLC candidates will therefore assist in developing effective therapeutic strategies for NSCLC.

Ropivacaine functions as both a monohydrate and anhydride, a unique amide-based local anesthetic characterized by its levorotatory nature [8]. Recent studies have noted that ropivacaine inhibits cancer progression in some cases. Ropivacaine inhibits the viability of GTPases in esophageal cancer cells [9], increases cellular apoptosis and restrains proliferation in liver cancer [10] and colon cancer [11]. In addition, ropivacaine can inhibit NSCLC cell growth [12] and induce apoptosis in NSCLC cells [13]. However, the specific regulatory mechanism of ropivacaine in NSCLC is little studied.

CircRNAs, a category of non-coding RNAs, are distinguished by a covalently sealed loop created via back-splicing [14]. The closed structure of circRNAs enhances their stability and resistance to RNA exonucleases [15]. The pathogenesis of NSCLC is influenced by circRNAs, according to recent studies [16, 17]. As well, some circRNAs act as microRNA (miRNA) inhibitors in NSCLC, thereby post-transcriptionally regulating gene expression [18–20]. In this study, high-throughput sequencing data from three normal lung tissues and three NSCLC tissues in the GSE112214 dataset were analyzed, and circ_0001320 expression was found to be downregulated in NSCLC. In addition, analysis of the bioinformatics website CircInteractome revealed a targeted binding site between circ_0001320 and miR-518a-5p. Therefore, we investigated whether ropivacaine modulated the circ_0001320/miR-518a-5p pathway to prevent NSCLC progression.

## Materials and methods

### Bioinformatics analysis

We analyzed high-throughput sequencing data from 3 normal lung samples and 3 NSCLC samples from the GSE112214 dataset for differentially expressed genes (DEGs) based on DESeq2 [21]. Screening for DEGs adhered to the edgeR filtering standards (log2 (fold change) > 2, false discovery rate > 0.05) [22]. DEGs that were upregulated and downregulated were classified based on log2 (Fold Change) > 1 and log2 (Fold Change) < − 1, respectively.

### Cell culture and treatment

Human NSCLC cell lines (H1299, H1975, and A549) and the normal lung epithelial cell line BEAS-2B were purchased from American Type Culture Collection (ATCC; USA). The cells were cultured in DMEM (Gibco, USA) supplemented with 10% fetal bovine serum (Invitrogen, USA) and 1% penicillin-streptomycin (Procell, Wuhan, China) in 5% CO_2_ at 37 °C. Ropivacaine (Sigma, USA) was dissolved by DMSO and diluted in PBS. sh-NC, sh-circ_0001320, mimic NC, miR-518a-5p mimic, sh-circ_0001320 + inhibitor NC or sh-circ_0001320 + miR-518a-5p inhibitor were constructed by RiboBio (Guangzhou, China). The above vectors (2 µg) or oligonucleotides (50 nM) were transfected into H1299 cells treated with ropivacaine (1 mmol/L) using Lipofectamine 2000 (Thermo Fisher, USA) [23].

### Tissue samples of patients

Tumor tissues and paracancerous normal tissues were collected from 40 NSCLC patients who were surgically resected in Longhua Hospital Shanghai University of Traditional Chinese Medicine. The experiment was approved by the Ethics Committee of Longhua Hospital Shanghai University of Traditional Chinese Medicine. Written informed consent was obtained from all patients. All procedures were carried out in accordance with relevant regulations and guidelines.

### RT-qPCR

Total RNA was extracted utilizing the TRIzol Reagent (Invitrogen). The creation of first strand cDNA utilized 1 µg of total RNA, employing Superscript II (Invitrogen, USA) for reverse transcription. RT-qPCR assays were conducted using Green Universal Master Mix reagent (Roche, USA) along with various primer mixtures. miR-518a-5p and circ_0001320 were normalized using U6 and GAPDH, respectively. Relative expression was calculated using the 2^−ΔΔCt^ method. The primers (Table 1) used for RT-qPCR analysis were purchased from Geneseed (Guangzhou, China).

**Table 1:** Primers

Genes	Primers
circ_0001320	Forward: 5’-GTGAAGCAGTGTGCGAAGA-3’
	Reverse: 5’-CTTGAGGTGTCATCATAGCCA-3’
miR-518a-5p	Forward: 5’-CTGCAAAGGGAAGCCCTT-3’
	Reverse: 5’-TATCCAGTGCGTGTCGTG-3’
U6	Forward: 5’-CTCGCTTCGGCAGCACA-3’
	Reverse: 5’-AACGCTTCACGAATTTGCGT-3’
GAPDH	Forward: 5’-CACCCACTCCTCCACCTTTG-3’
	Reverse: 5’-CCACCACCCTGTTGCTGTAG-3’

Note: miR-518a-5p, microRNA-518a-5p; GAPDH, Glyceraldehyde-3-phosphate dehydrogenase

### CCK-8

H1299 cells were distributed across 96-well plates, each well containing 2 × 10^4^ cells. Post 72 h, every well was added with 10 µl of CCK-8 reagent (Dojindo, Tokyo, Japan). Following a 2-hour period, 0.1% DMSO was introduced, and the absorbance was measured at 450 nm through a Bio-Rad microplate reader.

### Transwell assay

The study of migration utilized 24-Transwell inserts featuring non-coated membranes (8 μm pore size, Millipore), while the invasion examination employed matrigel-coated insert membranes (Millipore). Cells were moved to the upper chamber at 2 × 10^4^ per well for analyzing migration, and to the lower chamber at 1 × 10^5^ per well for examining invasion. Standard growth medium was used to fill the lower chamber. After a period of 24 h, the cells located on the bottom surface underwent staining using 0.2% crystal violet (Yeasen). Using Image J software, six views were analyzed under a Leica inverted microscope to determine the count of migrating or invading cells.

### Flow cytometry

The examination of apoptosis was conducted with the Annexin V-FITC/PI kit (Beyotime). H1299 cells were immersed in 100 µl binding buffer and conditioned to incubation with 10 µl of Annexin V-FITC and 10 µl of PI for a quarter-hour, isolated from light. To evaluate the rate of apoptosis, a BD FACSCalibur flow cytometer (BD Biosciences, USA) was employed.

### Tube formation assay

The culture supernatant of transfected NSCLC cells was added to the wells of 96-well plates that coated with Matrigel (BD Biosciences) and plated with human umbilical vein endothelial cells (HUVECs). Tube formation was observed under the microscope (Leica, Germany) after incubation at 37 °C for 8 h. The total tubular length per well was assessed via Image J software.

### Dual luciferase reporter gene assay

Predictions using the bioinformatics website CircInteractome (https://circinteractome.nia.nih.gov/) identified a targeted binding site between circ_0001320 and miR-518a-5p. The wild-type plasmid circ_0001320-WT and the mutant plasmid circ_0001320-MUT containing the miR-518a-5p binding site were integrated into the pGL3 promoter vector (GenePharma). After inoculation of H1299 cells into 24-well plates, circ_0001320-WT or circ_0001320-MUT was cotransfected into H1299 cells with miR-518a-5p mimic or mimic NC using Lipofectamine 2000 (Thermo Fisher), respectively. Finally, luciferase activity was assessed using a dual luciferase reporter gene assay kit (Promega).

### Data analysis

All data were processed using SPSS 21.0 statistical software, and measurements were expressed in the form of mean ± standard deviation. The t-test facilitated the comparison of measurements adhering to a normal distribution across two groups, whereas one-way ANOVA and Tukey’s post hoc tests were utilized for contrasting various groups. The statistical significance of the difference was identified by *p* < 0.05.

## Results

### Ropivacaine inhibits NSCLC cell proliferation, migration, and invasion and promotes apoptosis

**Fig. 1:**
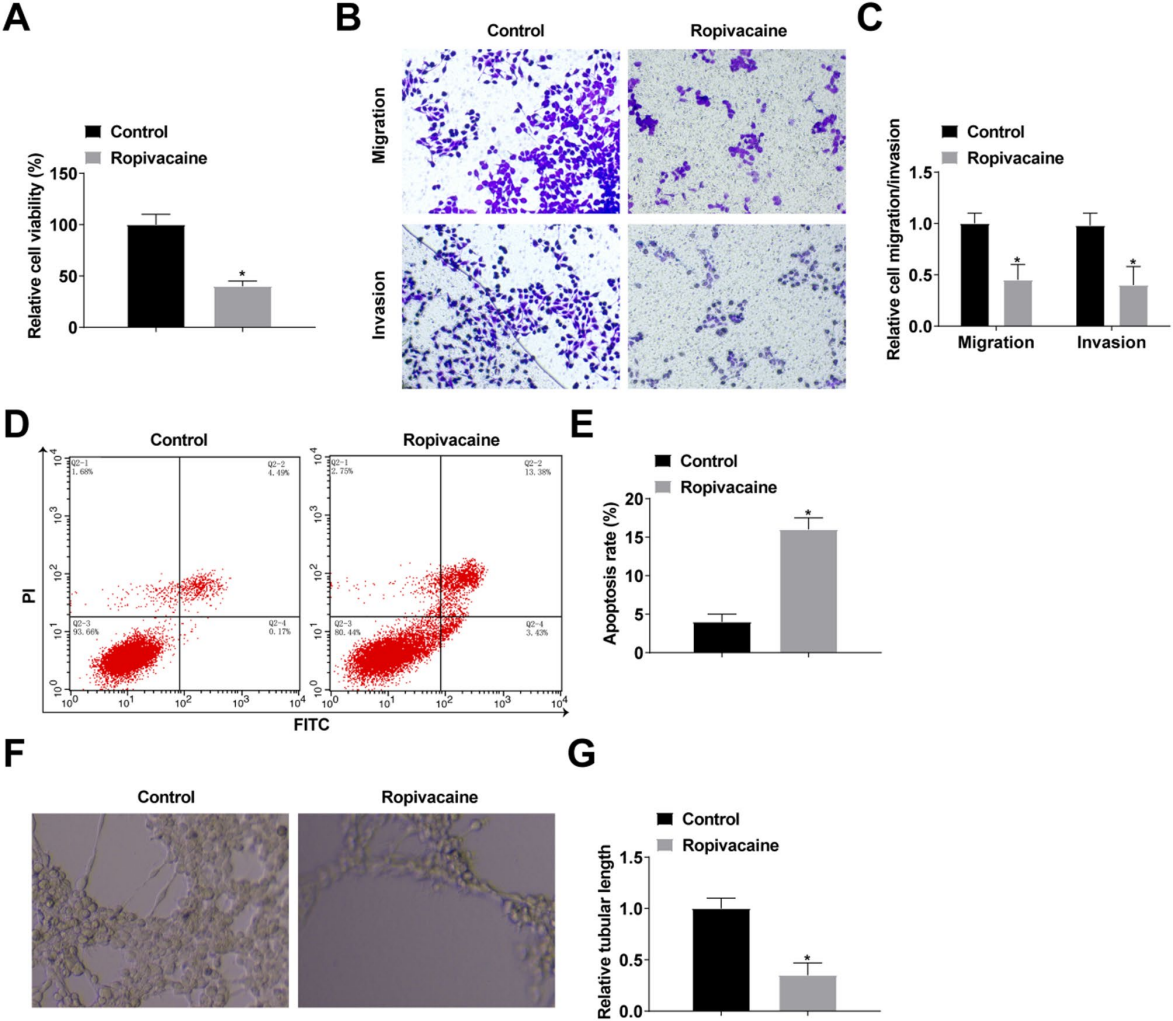
Ropivacaine inhibits NSCLC cell proliferation, migration and invasion and promotes apoptosis. **A**: Cell proliferation detected by CCK-8; **B/C**: Cell migration and invasion detected by transwell; **D/E**: Apoptosis detected by flow cytometry; **F/G**: Angiogenesis detected by tube formation assay. The data in the graphs are measures and expressed as mean ± standard deviation. * indicates *P* < 0.05 compared with Control group

To determine the regulatory effects of ropivacaine on NSCLC, we treated H1299 cells with 1 mmol/L ropivacaine. CCK-8 detection revealed that ropivacaine inhibited cell proliferation (Fig. 1A). As detected by transwell, ropivacaine inhibited cell migration and invasion (Fig. 1B, C). Flow cytometry analysis measured a increase in cellular apoptosis after ropivacaine treatment (Fig. 1D, E). Angiogenesis was detected by tube formation assay, which showed that ropivacaine inhibited angiogenesis (Fig. 1F, G).

### Reducing circ_0001320 attenuates the anti-NSCLC effect of ropivacaine

**Fig. 2:**
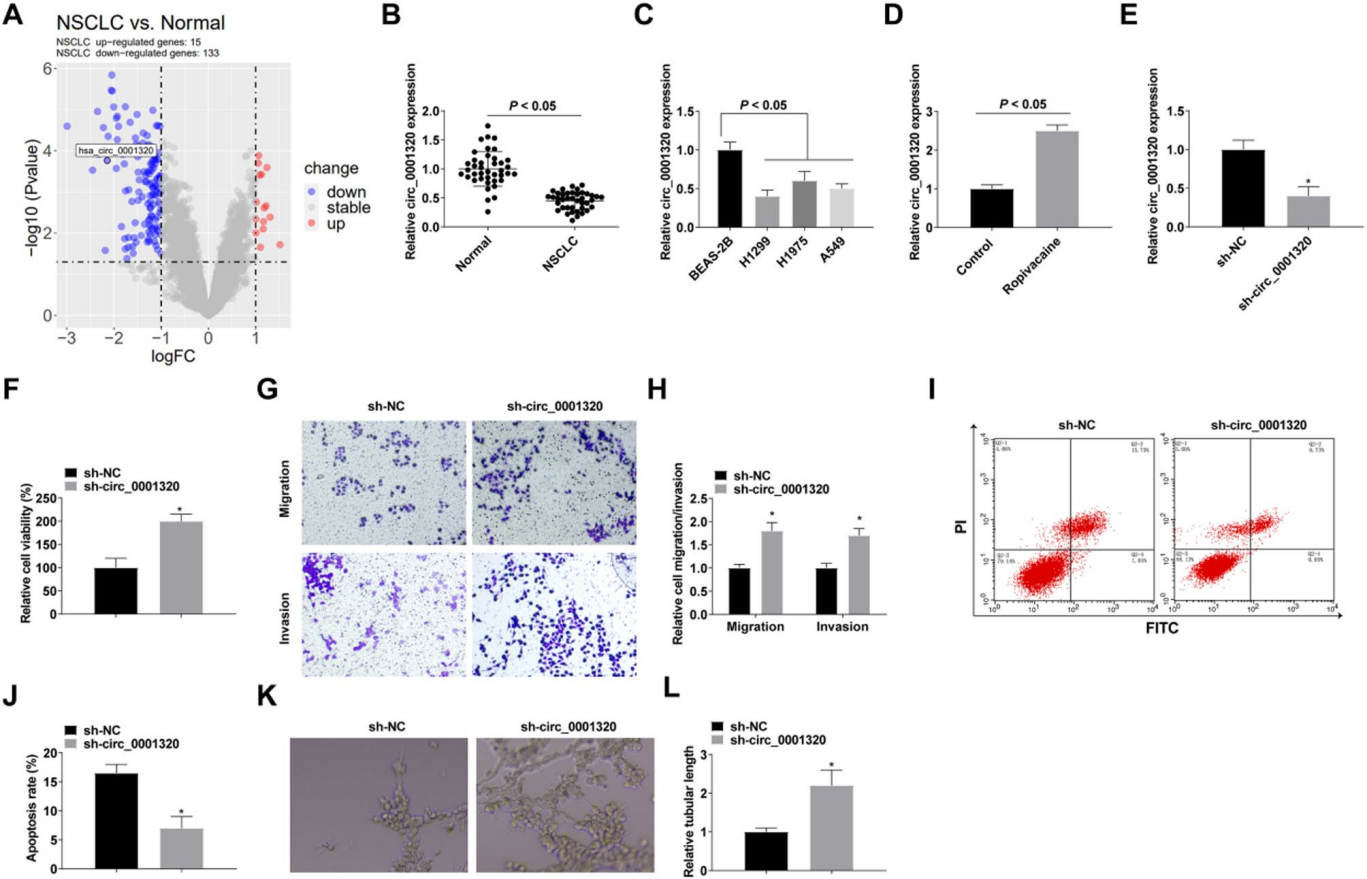
Down-regulation of circ_0001320 attenuates the inhibitory effect of ropivacaine on NSCLC progression. **A**: High-throughput sequencing data analysis; **B**: RT-qPCR detection of circ_0001320 expression in NSCLC tissues (*n* = 40); **C**: RT-qPCR detection of circ_0001320 expression in NSCLC cell lines; **D**: RT-qPCR detection of circ_0001320 expression after Ropivacaine treatment; **E**: RT-qPCR detection of circ_0001320 after transfection with sh-circ_0001320 (2 µg); **F**: CCK-8 detection of cell proliferation; **G/H**: Transwell detection of cell migration and invasion; **I/J**: Flow cytometry detection of cell apoptosis; K/L: Angiogenesis detected by tube formation assay. The data in the graphs are all measures and expressed as mean ± standard deviation. * indicates *P* < 0.05 compared with sh-NC group

Then, we further explored the downstream regulatory mechanisms of ropivacaine. Analysis of high-throughput sequencing data from three normal lung tissues and three NSCLC tissues in the GSE112214 dataset measured that circ_0001320 expression was downregulated in NSCLC (Fig. 2A). The expression of circ_0001320 in NSCLC tissues (*n* = 40) was then further verified, and it was found that circ_0001320 expression was downregulated in NSCLC tissues (Fig. 2B). It was also found that circ_0001320 expression was downregulated in NSCLC cell lines (Fig. 2C). And, ropivacaine could promote circ_0001320 expression (Fig. 2D). Therefore, we focused on circ_0001320 for subsequent studies.

By performing RT-qPCR on ropivacaine-treated H1299 cells, sh-NC and sh-circ_0001320 transfections were successfully verified (Fig. 2E). Reducing circ_0001320 was observed to lessen ropivacaine’s proliferation-inhibiting impact on H1299 cells (Fig. 2F). Concurrently, reducing circ_0001320 levels diminished ropivacaine’s impact on the migration and invasion of H1299 cells (Fig. 2G, H), along with those concerning apoptosis (Fig. 2I, J). Down-regulation of circ_0001320 attenuated the inhibitory effect of ropivacaine on angiogenesis (Fig. 2K, L).

### Circ_0001320 targets miR-518a-5p and inhibits its expression

**Fig. 3:**
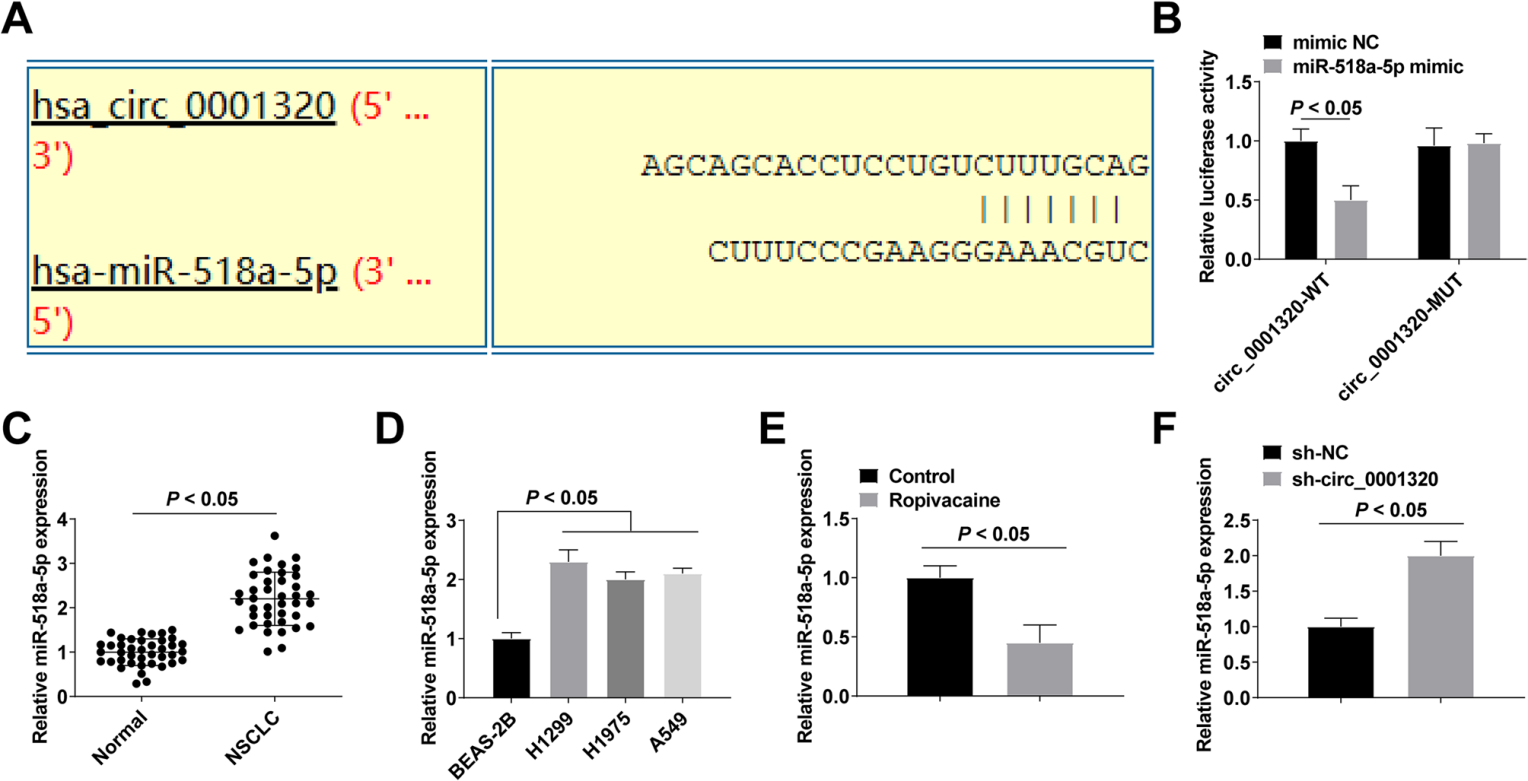
Circ_0001320 targets miR-518a-5p and inhibits its expression. **A**: Binding site of circ_0001320 and miR-518a-5p; **B**: Dual luciferase digestion reporter assay to verify the targeting relationship between circ_0001320 and miR-518a-5p; **C**: RT-qPCR detection of miR-518a-5p expression in NSCLC tissues (*n* = 40); **D**: RT-qPCR detection of miR-518a-5p expression in NSCLC cell lines; E: After ropivacaine treatment, RT-qPCR detection of miR-518a-5p expression; **F**: After down-regulation of circ_0001320, RT-qPCR detection of miR-518a-5p expression. The data in the graphs are all measures and expressed as mean ± standard deviation

The next step was to analyze in detail the miRNAs that may bind to circ_0001320. Analysis by the bioinformatics website CircInteractome revealed a targeted binding site between circ_0001320 and miR-518a-5p (Fig. 3A). To confirm this, we performed a dual luciferase reporter plasmid assay, which showed that miR-518a-5p mimic reduced the luciferase activity of the circ_0001320-WT (Fig. 3B), suggesting a targeting relationship between circ_0001320 and miR-518a-5p. In addition, miR-518a-5p expression was upregulated in NSCLC tissues and cell lines (Fig. 3C, D). Ropivacaine inhibited miR-518a-5p expression, while down-regulating circ_0001320 reduced this expression inhibition (Fig. 3E, F).

### Upregulation of miR-518a-5p accelerates ropivacaine-regulated NSCLC progression

**Fig. 4:**
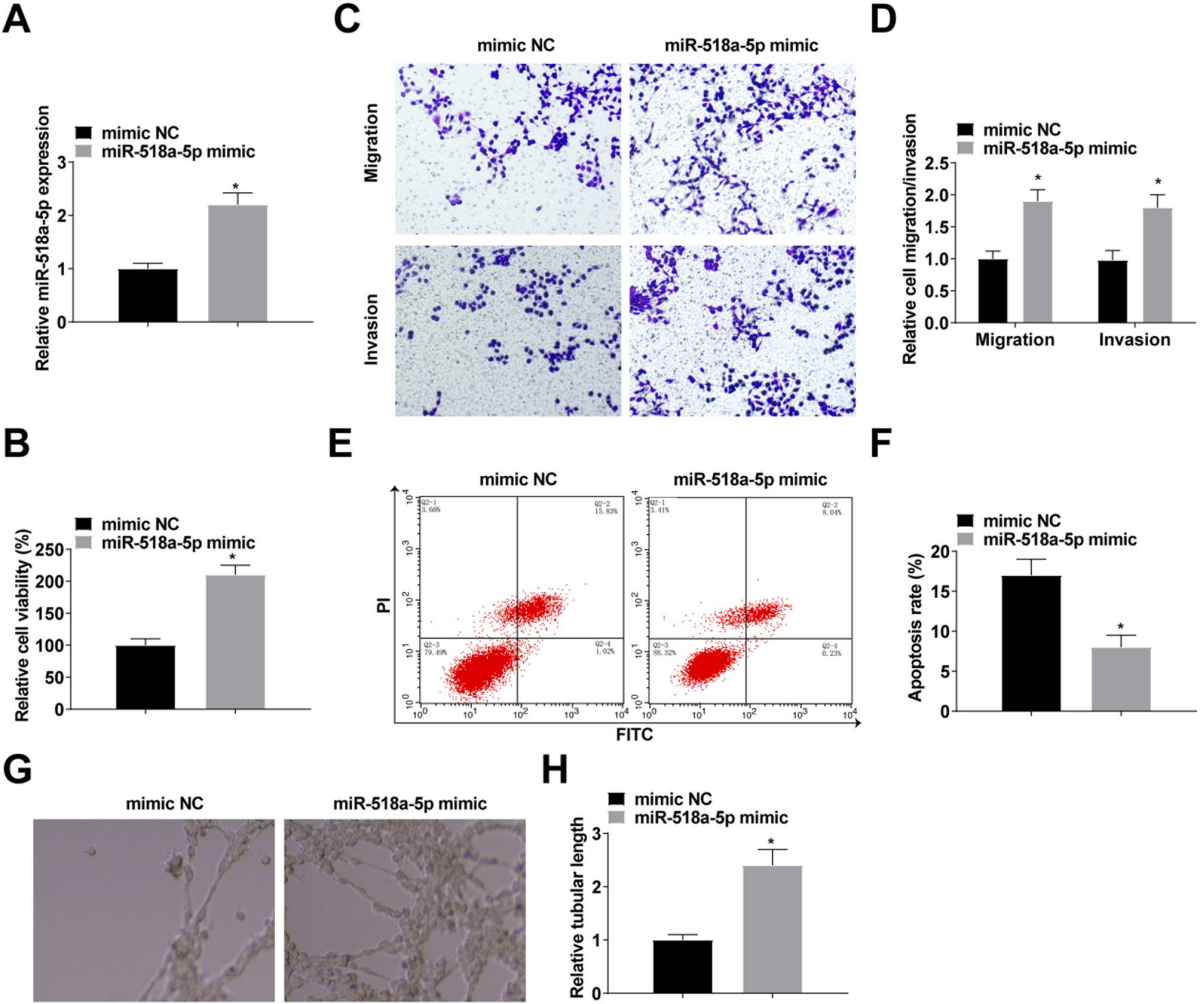
Up-regulation of miR-518a-5p attenuates the inhibitory effect of ropivacaine on NSCLC progression. **A**: RT-qPCR detection of miR-518a-5p after transfection with miR-518a-5p mimic (50 nM); **B**: CCK-8 for cell proliferation; **C/D**: Transwell for cell invasion; **E/F**: Flow cytometry for apoptosis; **G/H**: Angiogenesis detected by tube formation assay. The data in the graphs are all measures and expressed as mean ± standard deviation. * indicates *P* < 0.05 compared with mimic NC group

We investigated the role of miR-518a-5p in NSCLC by transfecting ropivacaine-treated H1299 cells with mimic NC and miR-518a-5p mimic, confirming transfection success via RT-qPCR (Fig. 4A). Research findings indicated that enhancing miR-518a-5p diminished ropivacaine’s tumor-fighting impact on H1299 cell growth (Fig. 4B-F). Upregulation of miR-518a-5p attenuated the inhibitory effect of ropivacaine on angiogenesis (Fig. 4G, H).

### Circ_0001320/miR-518a-5p axis is involved in NSCLC progression

**Fig. 5:**
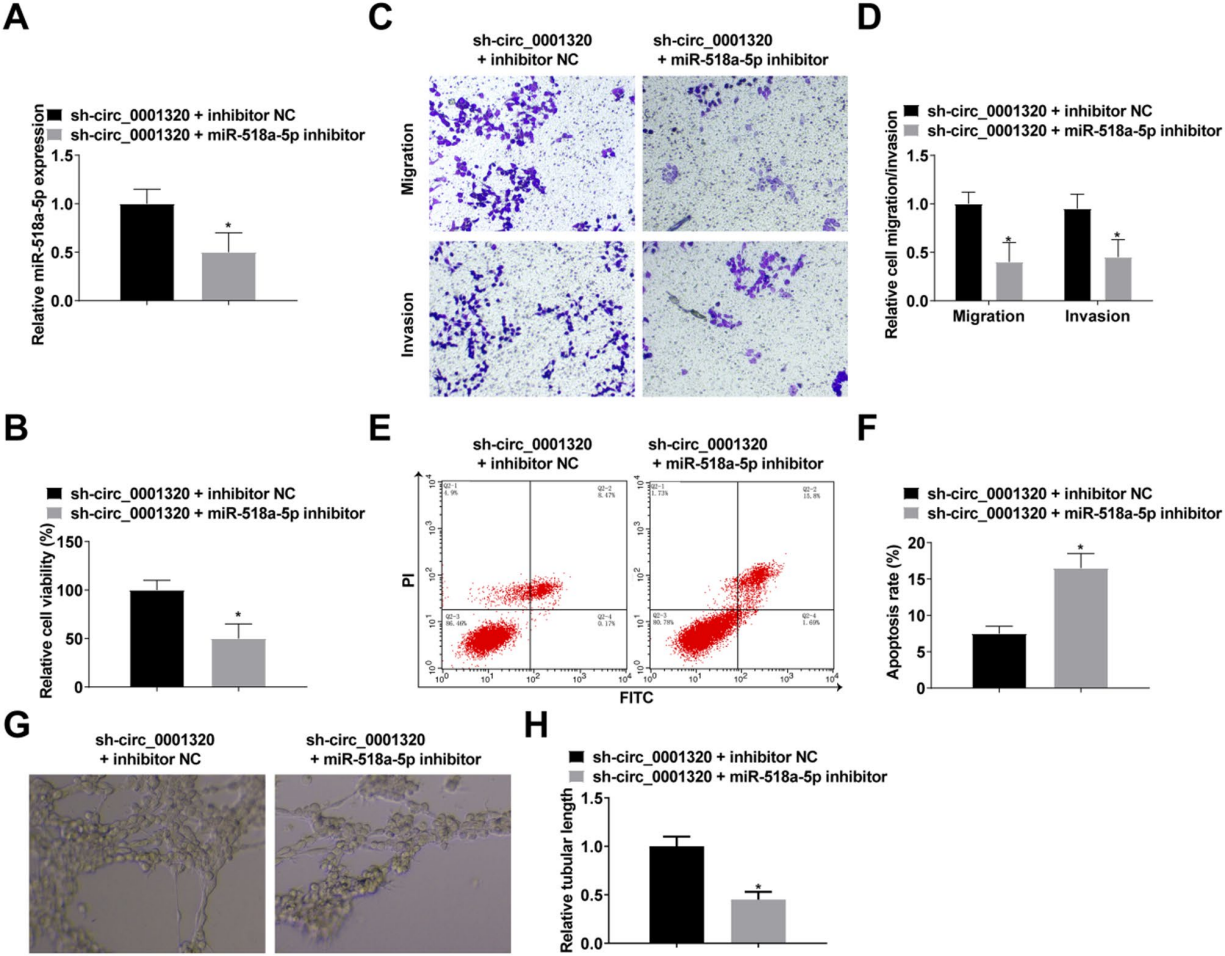
Down-regulation of miR-518a-5p reverses the effect of down-regulation of circ_0001320 on NSCLC progression. **A**: RT-qPCR detection of miR-518a-5p after transfection with miR-518a-5p inhibitor (50 nM); **B**: CCK-8 to detect cell proliferation; **C/D**: Transwell to detect cell migration and invasion; **E/F**: Flow cytometry to detect cell apoptosis; **G/H**: Angiogenesis detected by tube formation assay. The data in the graphs are all measures and expressed as mean ± standard deviation. * indicates *P* < 0.05 compared with sh-circ_0001320 + inhibitor NC group

Finally, we verified the involvement of the circ_0001320/miR-518a-5p axis in NSCLC by transfecting sh-circ_0001320 + inhibitor NC or sh-circ_0001320 + miR-518a-5p inhibitor into ropivacaine-treated H1299 cells. By RT-qPCR, the transfection was confirmed to have been successful (Fig. 5A). The results detailed that down-regulation of miR-518a-5p rescued the promoting effect of circ_0001320 silencing on H1299 cells (Fig. 5B-F). Downregulation of miR-518a-5p reversed the effect of downregulation of circ_0001320 on angiogenesis (Fig. 5G, H).

## Discussion

This study investigated the role and mechanism of ropivacaine in NSCLC progression, with the following results: (1) ropivacaine inhibited NSCLC proliferation, migration, invasion and angiogenesis, and promoted apoptosis; (2) ropivacaine promoted the expression of circ_0001320, and downregulation of circ_0001320 attenuated the inhibitory effect of ropivacaine on NSCLC progression; (3) circ_0001320 targets miR-518a-5p and inhibits its expression; (4) upregulation of miR-518a-5p attenuates the inhibitory effect of ropivacaine on NSCLC progression; (5) downregulation of miR-518a-5p reversed the effect of downregulation of circ_0001320 on NSCLC progression. In conclusion, the present study demonstrated for the first time that ropivacaine inhibited NSCLC progression by modulating the circ_0001320/miR-518a-5p axis.

The anesthetic ropivacaine exhibits antitumor properties. It suppresses miR-96/MEG2/pSTAT3 signaling in cervical cancer cells [24] and inhibits cancer angiogenesis by enhancing mitochondrial dysfunction and oxidative stress through sodium-channel-independent mitochondria [25]. Moreover, gastric cancer development is inhibited by ropivacaine by down-regulating ERK1/2 phosphorylation [26]. Papillary thyroid cancer cells are inhibited from migrating, invading, and proliferating because ropivacaine inhibits ITGA2 activation [27]. Ropivacaine inhibits gastric cancer invasion, migration, and growth by downregulating PI3K/AKT and WEE1 via miR-520a-3p [28]. Our analysis reported that NSCLC cells were suppressed in proliferation, migration, invasion, angiogenesis, and promoted in apoptosis by ropivacaine.

Tumor initiation and progression are closely associated with aberrant expression of non-coding RNAs [29]. There has been a recent boom in research on circRNA in ncRNA biology. In the past, CircRNAs were regarded as abnormally spliced transcripts with limited functions [30, 31]. The study of circRNAs through RNA sequencing and bioinformatics has shown their role in controlling gene expression in eucaryotes, with numerous circRNAs potentially influencing tumor development [32]. CircRNAs, with their distinct molecular composition and tissue-specific expression, stand as promising targets for therapeutic drugs and biomarkers in early cancer detection [33]. In this study, by analyzing high-throughput sequencing data from 3 normal lung tissues and 3 NSCLC tissues, circ_0001320 expression was downregulated in NSCLC tissues. We also discovered that down-regulating circ_0001320 attenuated the anti-tumor effects of ropivacaine on NSCLC cells.

CircRNAs regulate gene expression by acting as ceRNAs to sponge miRNAs [34, 35]. Several studies have demonstrated the involvement of the circRNA/miRNA axis in NSCLC, such as circUSP7/miR-934 [36], circRNA_103993/miR-1271 [37], and circRNA_100565/miR-337-3p [38]. The circular interactome database was screened and miR-518a-5p was identified as a possible target of circ_0001320. Moreover, ropivacaine inhibited miR-518a-5p expression by up-regulating circ_0001320, and restoring miR-518a-5p attenuated the suppressive role of ropivacaine for NSCLC cells. Furthermore, down-regulation of miR-518a-5p reversed the promoting effect of down-regulation of circ_0001320 on NSCLC progression.

However, this study has limitations. First, this study was performed in only one NSCLC cell line (H1299); second, no animal experiments were performed in this study; third, the downstream regulatory mechanisms of miR-518a-5p were not further explored; and lastly, down-regulation of circ_0001320 and up-regulation of miR-518a-5p could not completely reverse ropivacaine-reduced NSCLC cell proliferation, suggesting that ropivacaine may also inhibit NSCLC progression through other mechanisms.

## Conclusion

In conclusion, ropivacaine inhibits the malignant properties of NSCLC cell via the circ_0001320/miR-518a-5p axis, suggesting its potential as an anti-cancer agent in NSCLC.

## Data Availability

The datasets used and/or analyzed during the present study are available from the corresponding author on reasonable request.
